# Hypoxia and aging: molecular mechanisms, diseases, and therapeutic targets

**DOI:** 10.1002/mco2.786

**Published:** 2024-10-15

**Authors:** Ayesha Nisar, Sawar Khan, Wen Li, Li Hu, Priyadarshani Nadeeshika Samarawickrama, Naheemat Modupeola Gold, Meiting Zi, Sardar Azhar Mehmood, Jiarong Miao, Yonghan He

**Affiliations:** ^1^ Key Laboratory of Genetic Evolution & Animal Models, KIZ/CUHK Joint Laboratory of Bioresources and Molecular Research in Common Diseases, Kunming Institute of Zoology Chinese Academy of Sciences Kunming Yunnan China; ^2^ Kunming College of Life Science University of Chinese Academy of Sciences Kunming China; ^3^ Key Laboratory of Healthy Aging Research of Yunnan Province, Kunming Institute of Zoology Chinese Academy of Sciences Kunming Yunnan China; ^4^ Department of Cell Biology, School of Life Sciences Central South University Changsha Hunan China; ^5^ Institute of Molecular Biology and Biotechnology The University of Lahore Lahore Pakistan; ^6^ Department of Endocrinology The Second Affiliated Hospital of Dali University (the Third People's Hospital of Yunnan Province) Kunming Yunnan China; ^7^ Department of Zoology Hazara University Mansehra Pakistan; ^8^ Department of Gastroenterology The First Affiliated Hospital of Kunming Medical University Kunming Yunnan China

**Keywords:** age‐related disease, aging, hypoxia, mechanism, therapeutic target

## Abstract

Aging is a complex biological process characterized by the gradual decline of cellular functions, increased susceptibility to diseases, and impaired stress responses. Hypoxia, defined as reduced oxygen availability, is a critical factor that influences aging through molecular pathways involving hypoxia‐inducible factors (HIFs), oxidative stress, inflammation, and epigenetic modifications. This review explores the interconnected roles of hypoxia in aging, highlighting how hypoxic conditions exacerbate cellular damage, promote senescence, and contribute to age‐related pathologies, including cardiovascular diseases, neurodegenerative disorders, cancer, metabolic dysfunctions, and pulmonary conditions. By examining the molecular mechanisms linking hypoxia to aging, we identify key pathways that serve as potential therapeutic targets. Emerging interventions such as HIF modulators, antioxidants, senolytics, and lifestyle modifications hold promise in mitigating the adverse effects of hypoxia on aging tissues. However, challenges such as the heterogeneity of aging, lack of reliable biomarkers, and safety concerns regarding hypoxia‐targeted therapies remain. This review emphasizes the need for personalized approaches and advanced technologies to develop effective antiaging interventions. By integrating current knowledge, this review provides a comprehensive framework that underscores the importance of targeting hypoxia‐induced pathways to enhance healthy aging and reduce the burden of age‐related diseases.

## Graphical abstract

Aging involves the decline of cellular functions and increased disease susceptibility. Hypoxia, or reduced oxygen availability, accelerates aging by influencing pathways like hypoxia‐inducible factors (HIFs), oxidative stress, and inflammation. This review highlights how hypoxia exacerbates cellular damage, promotes senescence, and contributes to age‐related diseases. Emerging therapies, such as HIF modulators and senolytics, show promise in mitigating hypoxia's impact on aging.

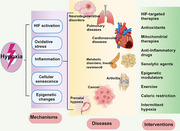

## 1 INTRODUCTION

Hypoxia, defined as reduced oxygen availability in tissues, is a physiological condition that can arise from various factors, including environmental exposure, disease states, and cellular dysfunction. Oxygen is vital for aerobic respiration, serving as the final electron acceptor in the mitochondrial electron transport chain, generating most cellular adenosine triphosphate (ATP).^1^ In response to low‐oxygen levels, eukaryotic cells activate adaptive mechanisms involving hypoxia‐inducible factors (HIFs) that regulate genes associated with angiogenesis, metabolism, and survival.^2^, ^3^ While hypoxia is not a universal or continuous condition for all individuals, its effects on cellular function are particularly relevant under pathological states and specific environmental conditions. Hypoxia's role in aging is complex, with both detrimental and, in controlled settings, potentially beneficial effects, such as preconditioning for stress resilience.^4^ However, chronic or uncontrolled hypoxia is increasingly recognized as a significant contributor to aging‐associated tissue damage and disease.^5^, ^6^, ^7^, ^8^

Aging is a multifaceted process characterized by the gradual decline of cellular and physiological functions, increased susceptibility to stress, and the onset of age‐related diseases.^9^ While aging is a complex process with multiple contributing factors, hypoxia plays a pivotal role in accelerating and exacerbating its manifestations, through various molecular pathways, including increased oxidative stress, chronic inflammation, and cellular senescence.^10^ Mitochondrial dysfunction, a hallmark of aging, is particularly sensitive to hypoxic stress, as reduced oxygen impairs mitochondrial respiration, leading to increased reactive oxygen species (ROS) production and subsequent damage to cellular components.^11^ Furthermore, chronic hypoxia can promote maladaptive changes, such as altered HIF signaling and persistent inflammation, which accelerate aging and contribute to conditions like cardiovascular and neurodegenerative diseases.^12^

Although hypoxia is not a primary cause of aging, it serves as a significant stressor that can exacerbate aging‐related cellular damage, particularly in disease contexts. Understanding the dual role of hypoxia, its beneficial effects under controlled conditions and its harmful impact in chronic or pathological scenarios, is essential for developing targeted therapeutic approaches. Hypoxia contributes to functional decline during aging, in part due to the complex interactions between HIF pathways and key signaling networks such as nuclear factor κB (NF‐κB), sirtuins, mechanistic target of rapamycin complex 1 (mTORC1), AMP‐activated protein kinase (AMPK), and UNC‐51‐like kinase 1 (ULK1).^13^, ^14^, ^15^ The interplay among these pathways under hypoxic conditions influences cellular metabolism, stress responses, and inflammatory processes, which are pivotal in driving the aging process.

Traditionally, research has focused on the effects of acute and chronic hypoxia and human adaptations to high‐altitude environments. However, recent studies have highlighted the detrimental effects of hypoxia, particularly in accelerating the progression of aging and metabolic disorders. This review aims to examine the negative impacts of hypoxia in the context of aging and disease, emphasizing the molecular mechanisms that mediate these effects and the potential therapeutic targets that could mitigate the associated risks. In the subsequent sections we explored these aspects.

## 2 MOLECULAR MECHANISMS LINKING HYPOXIA TO AGING

**FIGURE 1:**
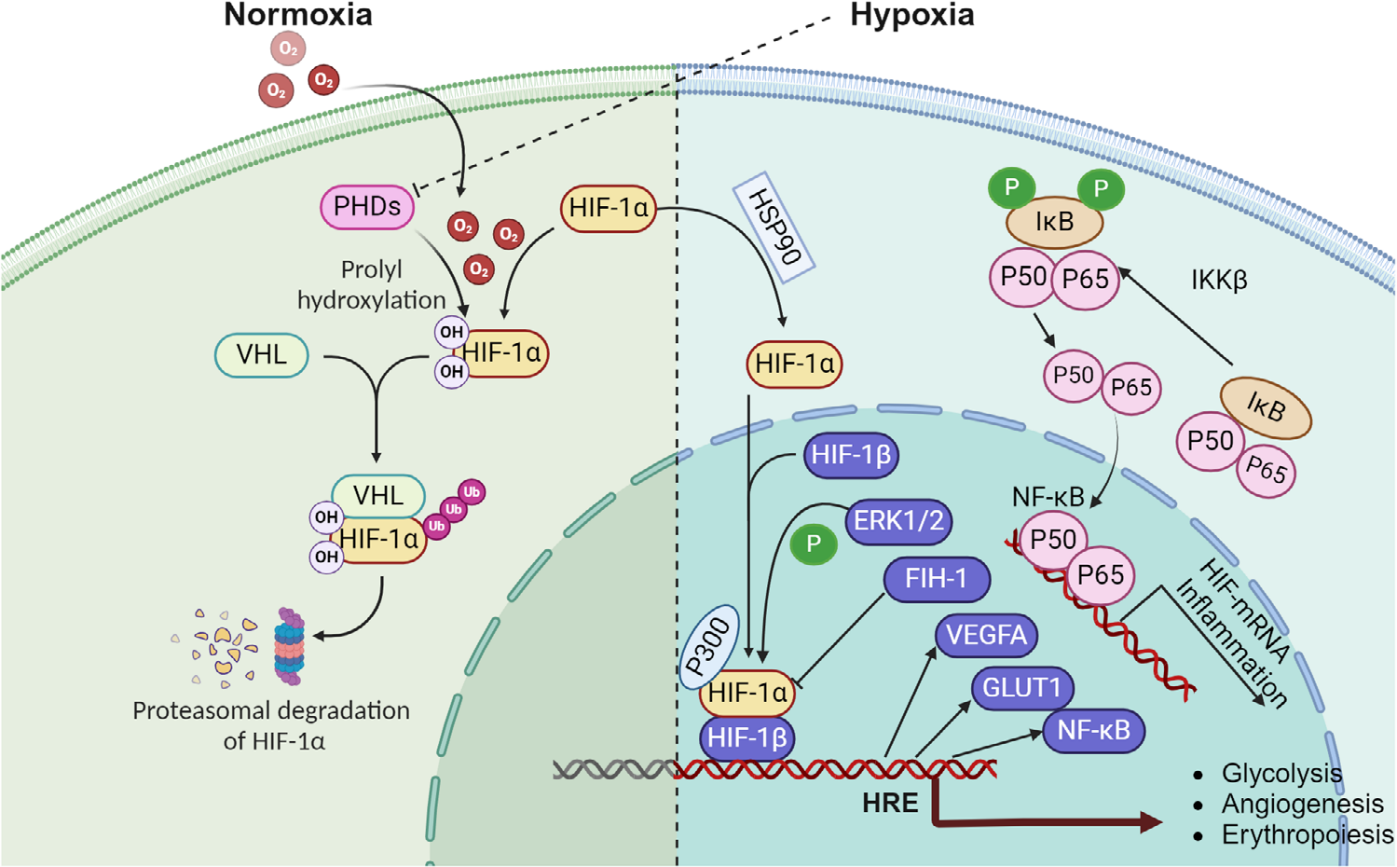
Overview of hypoxia‐inducible factors (HIFs) regulation. The activation of HIF‐1α under hypoxic condition is depicted. Under normal oxygen levels, the interaction between HIF‐1α and the von Hippel–Lindau (VHL) protein is oxygen‐dependent and involves the hydroxylation of specific proline residues on HIF‐1α by prolyl hydroxylases (PHDs). This hydroxylation targets HIF‐1α for ubiquitination by VHL, leading to its subsequent degradation via the proteasome. In low‐oxygen (hypoxic) conditions, hydroxylation of HIF‐1α is inhibited, preventing its degradation. This allows HIF‐1α to accumulate, dimerize with HIF‐1β, and bind to hypoxia response elements (HREs) on DNA, activating the transcription of genes that mediate adaptive cellular responses to hypoxia. ERK1/2, extracellular signal‐regulated kinase 1/2; FIH‐1, factor inhibiting hypoxia‐inducible factor 1; GLUT1, glucose transporter 1; HIF‐1α, hypoxia‐inducible factor 1‐alpha; HIF‐2α, hypoxia‐inducible factor 2‐alpha; IκB, inhibitor of nuclear factor kappa B; IKKβ, IκB kinase beta; NF‐κB, nuclear factor kappa B; PHDs, prolyl hydroxylases; P50, the 50‐kDa subunit of the NF‐κB transcription factor (also known as NF‐κB1); P65, the 65‐kDa subunit of the NF‐κB transcription factor (also known as RelA); VEGFA, vascular endothelial growth factor A.

**FIGURE 2:**
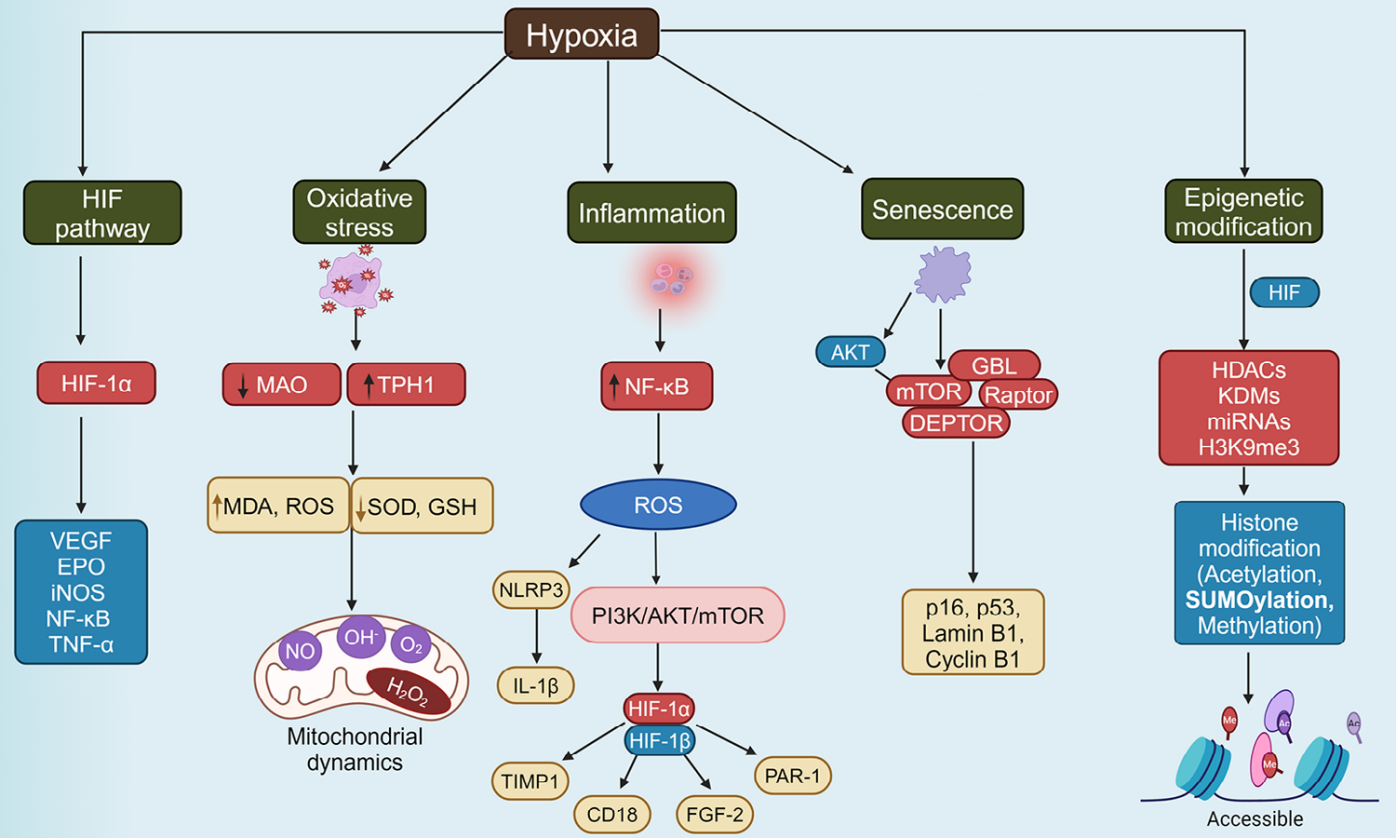
Molecular mechanisms underlying hypoxia‐induced aging. Hypoxia triggers a complex network of interconnected molecular pathways that contribute to aging and cellular dysfunction. Key mechanisms include oxidative stress, where an imbalance between reactive oxygen species (ROS) and antioxidant defenses leads to cellular damage; inflammation, characterized by the activation of proinflammatory signaling pathways that promote chronic inflammation; and cellular senescence, a state of permanent cell cycle arrest associated with the secretion of proinflammatory cytokines, growth factors, and matrix remodeling enzymes known as senescence‐associated secretory phenotype (SASP). Additionally, hypoxia induces epigenetic changes, such as DNA methylation, histone modification, and alterations in noncoding RNA expression, which impact gene expression and accelerate aging processes. Together, these mechanisms create a feedback loop that exacerbates cellular damage, impairs tissue function, and accelerates aging, contributing to age‐related diseases. CD18, integrin subunit beta‐2; EPO, erythropoietin; FGF‐2, fibroblast growth factor‐2; GBL, G protein beta subunit; GSH, glutathione; HDACs, histone deacetylases; HIF, hypoxia‐inducible factor; HIF‐1α, hypoxia‐inducible factor 1‐alpha; HIF‐1β, hypoxia‐inducible factor 1‐beta; IL‐1β, interleukin‐1 beta; iNOS, inducible nitric oxide synthase; KDMs, lysine demethylases; MAO, monoamine oxidase; MDA, malondialdehyde; NF‐κB, nuclear factor kappa B; NLRP3, NOD‐like receptor family pyrin domain containing‐3; PAR‐1, proteinase‐activated receptor‐1; ROS, reactive oxygen species; SOD, superoxide dismutase; TIMP1, metalloproteinase inhibitor‐1; TNFα, tumor necrosis factor‐alpha; TPH1, tryptophan hydroxylase‐1; VEGF, vascular endothelial growth factor.

Understanding the molecular mechanisms through which hypoxia influences aging is crucial for unraveling the complexities of age‐related decline. Hypoxia, characterized by reduced oxygen availability at the cellular level, triggers a cascade of molecular responses that significantly impact aging processes (Table 1). One of the primary pathways involved is the activation of HIFs (Figure 1), which regulate the expression of genes responsible for cellular metabolism, angiogenesis, and survival.^16^, ^17^ HIFs act as master regulators under low‐oxygen conditions, altering mitochondrial function, and promoting a shift from oxidative phosphorylation to glycolysis, which can lead to increased ROS production.^18^ This oxidative stress is compounded by impaired mitochondrial dynamics and mitophagy, contributing to the accumulation of damaged mitochondria—a hallmark of aging.^19^, ^20^ Additionally, hypoxia induces chronic inflammation and cellular senescence, two processes that accelerate the biological aging of tissues.^21^ The interplay between hypoxia, oxidative stress, inflammation, and epigenetic modifications creates a complex network of interactions that drive the aging process, laying the foundation for the onset of age‐related diseases. These molecular disruptions are further intensified by age‐related changes in the regulation of DNA repair and maintenance, with hypoxia contributing to genomic instability and telomere attrition.^22^, ^23^ Cellular senescence, often triggered by hypoxic stress, is a state of irreversible cell cycle arrest that contributes to tissue dysfunction and aging through the secretion of proinflammatory cytokines, chemokines, and proteases collectively known as the senescence‐associated secretory phenotype (SASP).^24^, ^25^ The epigenetic landscape is also profoundly affected by hypoxia, as DNA methylation and histone modifications under low‐oxygen conditions can lead to gene silencing or aberrant activation of aging‐associated pathways.^26^ Understanding these interconnected molecular mechanisms (Figure 2) is essential for identifying therapeutic targets that can mitigate the effects of hypoxia on aging and promote healthier aging trajectories.

**TABLE 1:** Molecular mechanisms of hypoxia‐induced aging and disease progression.

Molecular mechanism	Key players	Impact on aging and disease	References
Oxidative stress and mitochondrial dysfunction	ROS, mitochondria, antioxidants	Increases cellular damage, promotes aging, and contributes to neurodegeneration and cardiovascular diseases.	^11^, ^27^, ^28^
Inflammation and NF‐κB activation	NF‐κB, HIFs, TNF‐α, IL‐1β, IL‐6	Drives chronic inflammation, cellular senescence, and accelerates tumorigenesis.	^29^, ^30^
Epigenetic modifications	DNA methylation, histone acetylation, miRNAs	Alters gene expression, impairs neurodevelopment, and predisposes to neurodegenerative disorders.	^31^, ^32^
Hypoxia‐inducible factors (HIFs)	HIF‐1α, HIF‐2α, PHDs	Modulate immune response, oxidative stress, and influence cancer progression.	^33^, ^34^
Cellular senescence	p53, p21, SASP components	Promotes a proinflammatory environment, contributing to tissue aging and dysfunction.	^35^, ^36^, ^37^
Autophagy and proteostasis	ULK1, mTOR, AMPK, SIRT1	Impaired autophagy leads to the accumulation of damaged proteins, exacerbating aging.	^38^
Metabolic reprogramming	HIF‐1α, mTOR, PGC‐1α	Alters glucose metabolism and mitochondrial function, contributing to age‐related metabolic diseases.	^39^
Epigenetic clock dysregulation	DNA methylation clock	Accelerates aging phenotypes through changes in gene expression patterns.	^40^
DNA damage response	ATM, ATR, PARP	Persistent DNA damage signals induce cellular senescence and genomic instability.	^41^
Telomere shortening	Telomerase, shelterin complex	Leads to replicative senescence, limiting tissue regeneration.	^42^, ^43^
Altered cell signaling	Wnt, notch, TGF‐β	Dysregulation contributes to tissue fibrosis and impaired regeneration.	^44^
Impaired immune surveillance	NK cells, CD8^+^ T cells	Reduced immune function increases susceptibility to infections and cancer.	^45^
Fibrosis and extracellular matrix remodeling	TGF‐β, collagen, MMPs	Promotes tissue stiffening and loss of function, common in aging organs.	^46^

Abbreviations: AMPK, AMP‐activated protein kinase; ATM, ataxia telangiectasia mutated; ATR, ataxia telangiectasia and Rad3‐related; CD8^+^ T cells, CD8‐positive T cells; HIF‐1α, hypoxia‐inducible factor 1‐alpha; HIF‐2α, hypoxia‐inducible factor 2‐alpha; HIFs, hypoxia‐inducible factors; IL‐1β, interleukin‐1 beta; IL‐6, interleukin‐6; miRNAs, microRNAs; MMPs, matrix metalloproteinases; mTOR, mechanistic target of rapamycin; NF‐κB, nuclear factor kappa‐light‐chain‐enhancer of activated B cells; NK cells, natural killer cells; p21, cyclin‐dependent kinase inhibitor 1A (also known as CDKN1A); p53, tumor protein p53; PARP, poly (ADP‐ribose) polymerase; PGC‐1α, peroxisome proliferator‐activated receptor gamma coactivator 1‐alpha; PHDs, prolyl hydroxylases; ROS, reactive oxygen species; SASP, senescence‐associated secretory phenotype; SIRT1, sirtuin 1; TGF‐β, transforming growth factor‐beta; TNF‐α, tumor necrosis factor‐alpha; ULK1, Unc‐51 like autophagy activating kinase 1.

### 2.1 Hypoxia‐inducible factors and aging

HIFs, particularly HIF‐1α and HIF‐2α, are transcription factors that serve as master regulators of the cellular response to low‐oxygen levels. HIF‐1α is stabilized under hypoxic conditions, translocates to the nucleus, and activates the transcription of genes involved in angiogenesis, glucose metabolism, and erythropoiesis.^16^, ^47^ During aging, there is a marked decline in the adaptive capacity of HIF signaling, which has been linked to reduced cellular stress resilience, mitochondrial dysfunction, and impaired angiogenesis.^48^ Studies in animal models show that decreased HIF‐1α activity with age contributes to a decline in vascular and metabolic homeostasis, promoting age‐related diseases.^33^, ^34^

The crosstalk between HIFs and other key signaling pathways also plays a critical role in aging. For example, HIFs modulate mTOR and AMPK pathways, which are essential for cellular metabolism, autophagy, and stress response.^49^, ^50^ Dysregulation of these pathways by altered HIF activity in aging cells leads to metabolic imbalances, reduced autophagy, and increased susceptibility to oxidative stress.^51^ The reduced function of HIFs with age may also affect stem cell maintenance and regenerative capacity, contributing to tissue aging and dysfunction.^52^ Furthermore, HIFs play a crucial role in modulating mitochondrial function, a key aspect of cellular metabolism and aging.^53^ HIF‐1α regulates mitochondrial respiration by altering the expression of genes involved in the electron transport chain and glycolysis, shifting energy production from oxidative phosphorylation to glycolysis under hypoxic conditions.^54^ This metabolic reprogramming helps cells adapt to low‐oxygen levels but can also lead to increased ROS production and mitochondrial damage, especially in aging cells with compromised antioxidant defenses.^55^ The decline in HIF‐1α function with age exacerbates mitochondrial dysfunction, contributing to the accumulation of cellular damage and promoting aging.

Recent studies suggest that enhancing HIF‐1α activity may promote healthy aging by improving metabolic homeostasis, reducing oxidative stress, and enhancing angiogenesis.^56^, ^57^, ^58^ However, the therapeutic potential of targeting HIFs in aging remains controversial, as chronic HIF activation has also been linked to tumorigenesis and other pathological condition.^59^ Thus, a better understanding of the context‐dependent effects of HIF modulation is necessary for developing effective antiaging therapies.

### 2.2 Oxidative stress and mitochondrial dysfunction

Oxidative stress, defined as the imbalance between ROS production and antioxidant defense, is a fundamental mechanism driving aging and age‐related diseases. Hypoxia exacerbates oxidative stress by impairing mitochondrial respiration, leading to increased electron leakage and ROS generation.^11^ ROS can cause extensive damage to cellular macromolecules, including DNA, proteins, and lipids, resulting in cellular dysfunction, senescence, and death.^60^, ^61^ Under hypoxic conditions, mitochondrial dysfunction is further aggravated, contributing to the progressive decline in cellular and tissue function observed with aging.^27^, ^28^

Hypoxia also impacts mitochondrial dynamics, including fission, fusion, and mitophagy, processes essential for maintaining mitochondrial integrity and function.^62^ Hypoxia‐induced oxidative stress disrupts these dynamics, leading to the accumulation of damaged mitochondria, decreased ATP production, and increased apoptosis. This mitochondrial dysfunction is particularly pronounced in aging cells, where the capacity for mitophagy and mitochondrial biogenesis is already compromised, resulting in a vicious cycle of increased oxidative damage and cellular decline.^63^ Moreover, mitochondrial dysfunction under hypoxic conditions contributes to the activation of proinflammatory pathways, creating a feedback loop that promotes chronic inflammation and accelerates aging.^64^ The release of mitochondrial DNA and other damage‐associated molecular patterns (DAMPs) can activate the inflammasome and other innate immune responses, contributing to “inflammaging.”^65^, ^66^ Inhibition of mitochondrial ROS production or enhancement of mitochondrial quality control mechanisms has shown promise in delaying aging and extending lifespan in animal models.^67^, ^68^ Evidence from animal studies and clinical trials suggests that mitochondrial dysfunction and oxidative damage caused by excessive ROS production are major contributors to aging and age‐related diseases. Mitochondrial abnormalities have been observed in the epithelial and airway smooth muscle cells of patients with chronic obstructive pulmonary disease (COPD), indicating the role of mitochondrial impairment in the pathology of this condition.^69^ Recently, a study demonstrated that patients with COPD have significantly higher systemic inflammatory markers, such as high‐sensitivity C‐reactive protein (hs‐CRP) and leukocytes, and reduced levels of the antioxidant glutathione (GSH), compared to individuals without COPD.^70^

Moreover, excess ROS production can trigger ferroptosis, a type of cell death characterized by decreased cellular antioxidant capacity and ROS accumulation. Researchers have noted that this process is accompanied by substantial iron accumulation, lipid peroxidation, altered mitochondrial structure, somatic membrane wrinkling, and fragmentation of the outer membrane. Studies suggest that mitochondrial ROS are involved in regulating immune responses and autophagy‐related signaling pathways, such as the AMPK–ULK1 axis, which promotes iron autophagy.^71^ Additionally, elevated free iron levels have been shown to induce ROS, resulting in cellular damage and potential cell death.^72^ These findings underscore the critical role of mitochondrial ROS in age‐related conditions, influencing both cellular function and systemic health.

### 2.3 Inflammatory pathways activation

Chronic inflammation, also known as “inflammaging” is a hallmark of aging that is exacerbated by hypoxia. Hypoxia triggers the activation of various inflammatory pathways, including NF‐κB and HIF‐1, leading to the production of proinflammatory cytokines (Table 2) such as interleukin‐6 (IL‐6), tumor necrosis factor‐alpha (TNF‐α), and interleukin‐1 beta (IL‐1β).^29^, ^30^ These cytokines contribute to chronic low‐grade inflammation, which is implicated in the pathogenesis of numerous age‐related diseases, including cardiovascular disease (CVD), neurodegeneration, and cancer.^73^ The relationship between hypoxia and inflammation is complex and involves multiple feedback loops. For instance, hypoxia‐induced ROS can activate the NOD‐like receptor (NLR) family pyrin domain containing 3 (NLRP3) inflammasome, promoting the release of IL‐1β and further exacerbating inflammation.^74^, ^75^, ^76^ Additionally, hypoxia can induce the expression of adhesion molecules, such as ICAM‐1 and VCAM‐1, promoting leukocyte recruitment and perpetuating the inflammatory response.^77^ The chronic activation of these inflammatory pathways under hypoxic conditions is implicated in the induction of tissue damage, promotion of cellular senescence, and acceleration of the aging process.

**TABLE 2:** Hypoxia‐induced inflammatory responses in aging and disease contexts.

Condition	Inflammatory markers	Effect of hypoxia	Clinical implications	References
Acute mountain sickness (AMS)	IL‐6, IL‐1β, CRP	Elevated during hypoxic exposure, disrupts blood–brain barrier.	Contributes to AMS symptoms and cerebral edema.	^99^, ^100^, ^101^, ^102^, ^103^
COPD	TAT, D‐dimers, VWF	Increases coagulation and inflammatory responses.	Heightens risk of vascular complications in COPD.	^104^, ^105^
Colorectal cancer	TNF‐α, NF‐κB	Promotes tumor progression and angiogenesis.	Linked to worsened prognosis and tumor growth.	^106^
Neurodegeneration (Alzheimer's)	S100A8, IL‐6, TNF‐α	Induces neuroinflammation and neuronal apoptosis.	Contributes to cognitive decline and AD pathology.	^107^, ^108^, ^109^
High‐altitude cerebral edema (HACE)	IL‐6, AQP4	Enhances cerebral edema through blood–brain barrier disruption.	Increases severity of altitude sickness.	^99^, ^110^
High‐altitude pulmonary edema (HAPE)	CRP, TNF‐α	Leads to pulmonary hypertension and fluid buildup.	Impairs lung function and oxygen delivery.	^111^, ^112^
Non–small‐cell lung cancer (NSCLC)	Gr‐1^+^ granulocytic cells, CD45^+^ cells	HIF‐2α regulates immune cell infiltration, impacting tumor growth.	Modulates tumor immune microenvironment.	^113^
Pancreatic cancer (PDAC)	IL‐10, B Cells	Loss of HIF‐1α enhances tumor progression and immune cell infiltration.	Alters immune dynamics in tumor growth.	^114^, ^115^
High‐altitude pulmonary hypertension (HAPH)	IL‐1β, IL‐6	Promotes vascular remodeling and increased arterial pressure.	Leads to chronic hypoxic stress in high‐altitude residents.	^111^, ^112^
Obstructive sleep apnea (OSA)	IL‐6, TNF‐α, CRP	Intermittent hypoxia triggers systemic inflammation and oxidative stress.	Linked to cardiovascular diseases and cognitive decline.	^116^, ^117^, ^118^
Chronic kidney disease (CKD)	TNF‐α, IL‐1β	Enhances renal inflammation, contributing to disease progression.	Impairs kidney function and increases fibrosis.	^119^
Alzheimer's disease (AD)	Aβ, Tau, S100A8	Hypoxia‐induced inflammation worsens neurodegeneration.	Accelerates cognitive decline and neuronal death.	^5^
Cancer cachexia	IL‐6, TNF‐α, IL‐1β	Hypoxia exacerbates systemic inflammation, leading to muscle wasting.	Reduces quality of life in cancer patients.	^120^
Cardiovascular diseases	IL‐6, MCP‐1, CRP	Hypoxia increases vascular inflammation, contributing to atherosclerosis.	Heightens risk of heart attacks and strokes.	^121^, ^122^
Diabetes mellitus	IL‐1β, IL‐6, TNF‐α	Promotes insulin resistance and chronic inflammation in hypoxic tissues.	Worsens glucose metabolism and vascular health.	^123^, ^124^

Abbreviations: Aβ, amyloid beta; AQP4, aquaporin 4; B cells, B lymphocytes; CD45^+^, CD45 positive (leukocytes); COPD, chronic obstructive pulmonary disease; CRP, C‐reactive protein; D‐dimers, D‐dimer (a fibrin degradation product); Gr‐1^+^, Gr‐1 positive (granulocytic cells); IL‐1β, interleukin‐1 beta; IL‐6, interleukin‐6; IL‐10, interleukin‐10; MCP‐1, monocyte chemoattractant protein‐1; NF‐κB, nuclear factor kappa‐light‐chain‐enhancer of activated B cells; S100A8, S100 calcium‐binding protein A8; TAT, thrombin–antithrombin complex; TNF‐α, tumor necrosis factor‐alpha; VWF, von Willebrand factor.

Hypoxia‐induced activation of NF‐κB involves multiple pathways, including the roles of oxygen sensors, hydroxylases, JmjC enzymes, and kinases like IκB kinase (IKK) and transforming growth factor‐β (TGF‐β)‐activated kinase 1 (TAK1). Among the oxygen sensors, 2OG‐dependent dioxygenases, such as prolyl hydroxylases (PHDs) and factor inhibiting HIF (FIH), are crucial in stabilizing HIF and influencing pathways related to oxygen availability, including NF‐κB. Proteomics studies have identified components of the NF‐κB pathway, such as p105, IκBα, and OTU domain‐containing ubiquitin aldehyde‐binding proteins 1 (OTUB1), as hydroxylation targets of FIH, linking oxygen sensing to NF‐κB regulation.^78^, ^79^ Although OTUB1 hydroxylation affects cellular metabolism, its direct involvement in hypoxia‐induced NF‐κB activation is yet to be fully understood. PHDs also modulate NF‐κB activity by hydroxylating key regulators, including IKKβ, which is vital for NF‐κB activation under hypoxic conditions.^80^, ^81^, ^82^, ^83^, ^84^ JmjC enzymes, typically recognized for their role in demethylating lysine residues, also influence NF‐κB signaling by targeting nonhistone proteins such as p65. For instance, lysine demethylase 2A (KDM2A) demethylates p65, thereby regulating NF‐κB target gene expression. Notably, KDM2A is induced by both NF‐κB and hypoxia, establishing a feedback loop that connects NF‐κB signaling with hypoxic responses.^85^, ^86^, ^87^ Other JmjC enzymes, including lysine demethylase 6B (KDM6B) and jumonji domain containing 8 (JMJD8), further modulate NF‐κB signaling, illustrating the intricate crosstalk between hypoxia and inflammation pathways. Hypoxia also activates NF‐κB through IKK‐dependent pathways involving phosphorylation of IκBα, facilitated by TAK1 and ubiquitin‐conjugating enzyme Ubc13, and conserved across different species, emphasizing their fundamental role in hypoxic responses.^88^, ^89^ The involvement of ubiquitin ligases, such as X‐linked inhibitor of apoptosis (XIAP), in interacting with IKK further underscores the complexity of NF‐κB regulation under hypoxic conditions. Under hypoxic conditions, IκBα undergoes unique post‐translational modifications, such as SUMOylation instead of degradation, which distinctly influences NF‐κB activity compared to normoxic conditions.^90^ SUMOylation of IκBα can either promote or inhibit NF‐κB activation depending on the SUMO isoform involved, reflecting a nuanced, context‐dependent mechanism of NF‐κB modulation in hypoxia.

Cellular senescence itself is both a consequence and a driver of inflammation. Senescent cells secrete a range of SASP factors which can be enhanced by hypoxia, contributing to a proinflammatory environment that accelerates tissue aging and dysfunction.^91^, ^92^ Hypoxia‐induced inflammation has been confirmed in both clinical and experimental settings. A study from the University of Edinburgh reported hypoxemia and monocytopenia in patients with acute respiratory distress syndrome (ARDS) within 48 h of ventilation, which was also observed in mouse models of hypoxic acute lung injury, leading to reduced monocyte‐derived macrophages and increased neutrophil‐driven inflammation.^93^ In respiratory viral infections, such as influenza and COVID‐19, hypoxemia frequently occurs due to the interplay between lung inflammation and a hypoxic microenvironment that disrupts pulmonary function.^94^ Hypoxia is also implicated in promoting tissue inflammation and fibrosis by enhancing profibrotic cytokine responses, reducing antigen presentation, and suppressing T‐cell activity, particularly in nodular diseases.^95^ HIF‐1α and HIF‐2α play distinct roles in inflammation; HIF‐1α supports cardiomyocyte protection via macrophage glycolysis reprogramming, while HIF‐2α inhibits mitochondrial metabolism in anti‐inflammatory macrophages.^96^ Hypoxia activates NLRP3 inflammasomes, triggering IL‐1β release and inflammation in macrophages.^74^ In the nervous system, hypoxia‐induced S100A8 protein promotes neuroinflammation and apoptosis via JNK and ERK pathways.^97^ Additionally, intermittent hypoxia in tumors enhances a proinflammatory macrophage phenotype through the JNK/p65 pathway, highlighting the link between hypoxia and immune dysregulation.^98^ These findings underscore the complex relationship between hypoxia and inflammation, emphasizing the need to further explore hypoxia‐driven inflammatory signaling for therapeutic advancements.

### 2.4 Cellular senescence and DNA damage

Cellular senescence is a state of irreversible growth arrest that occurs in response to various stressors, including hypoxia and DNA damage. Senescent cells accumulate with age and contribute to tissue dysfunction and aging by secreting inflammatory factors and degrading the extracellular matrix.^35^, ^36^ Hypoxia has been shown to induce cellular senescence by promoting DNA damage through increased ROS production and impairing DNA repair mechanisms. This DNA damage response (DDR) triggers the activation of p53 and p21, key regulators of cell cycle arrest and senescence.^125^, ^126^

Hypoxia‐induced DNA damage occurs via direct and indirect mechanisms. Directly, hypoxia increases the production of ROS, leading to oxidative damage to DNA, proteins, and lipids.^127^ Indirectly, hypoxia downregulates key DNA repair pathways, such as homologous recombination and nonhomologous end joining, resulting in genomic instability and the accumulation of mutations.^128^, ^129^ This genomic instability contributes to cellular senescence and the development of age‐related diseases, including cancer. Furthermore, hypoxia‐induced senescence is associated with telomere attrition, where repeated exposure to low‐oxygen levels accelerates telomere shortening, a key marker of cellular aging.^130^ Telomere dysfunction activates the p53/p21 and p16^INK4a^ pathways, driving cells into a senescent state and contributing to aging and age‐related diseases.^131^ Inhibiting telomere shortening or enhancing telomerase activity has been proposed as a potential strategy for mitigating the effects of hypoxia on aging.^132^, ^133^

### 2.5 Epigenetic modifications in hypoxic aging

Epigenetic changes, including DNA methylation, histone modifications, and noncoding RNA regulation, play a crucial role in aging and are profoundly affected by hypoxia. Hypoxic conditions can lead to widespread epigenetic reprogramming (Table 3), impacting gene expression patterns associated with aging.^134^ For instance, hypoxia‐induced HIF‐1α stabilization can modulate the expression of various genes involved in metabolism, angiogenesis, and cellular survival.^135^, ^136^ Hypoxia has been shown to cause DNA hypermethylation and histone modifications that alter chromatin structure and function, promoting the expression of proaging genes while silencing genes involved in DNA repair and cell cycle control.^137^, ^138^, ^139^ Additionally, hypoxia can influence noncoding RNAs, such as microRNAs and long noncoding RNAs, which regulate gene expression at the post‐transcriptional level and contribute to aging.^31^, ^32^ Recent studies suggest that reversing epigenetic changes associated with hypoxia may offer therapeutic potential in delaying aging and treating age‐related diseases.^140^ However, the exact mechanisms by which hypoxia modulates epigenetic landscapes remain to be fully elucidated, requiring further research.

**TABLE 3:** Epigenetic modifications induced by hypoxia in aging and diseases.

Epigenetic modification	Affected genes/proteins	Impact	Relevant conditions	References
DNA methylation	Glucocorticoid receptors, APP	Alters gene expression, increases risk of cognitive deficits.	Prenatal hypoxia, Alzheimer's disease	^154^
Histone modifications	Histone acetylation, H3 methylation	Modifies chromatin structure, affects brain and lung development.	High‐altitude hypoxia, neurodevelopment	^139^
miRNA regulation	miR‐210, miR‐23b‐27b	Modulates neuronal apoptosis, influences neurodegenerative phenotypes.	Hypoxic brain injury, neurodegeneration	^148^, ^149^
Hydroxylation by dioxygenases	NF‐κB, IKKβ, OTUB1	Regulates inflammatory signaling pathways under hypoxia.	Inflammatory diseases, cancer	^79^
Methylation changes in hypoxia	HIF‐1α, p53	Dysregulated gene silencing contributes to cancer progression.	Colorectal cancer, breast cancer	^106^, ^155^
Chromatin remodeling	BRD4, SWI/SNF complex	Alters accessibility of transcription factors, affecting cell cycle and apoptosis.	Cancer, aging cells	^156^
Epigenetic clock	Horvath clock, Hannum clock	Predicts biological age based on DNA methylation patterns.	Aging, longevity research	^157^
Noncoding RNAs	lncRNAs, circRNAs	Regulate gene expression post‐transcriptionally, influencing aging phenotypes.	Tumor	^158^
Stress‐induced epigenetic changes	HSP70, HSP90	Heat shock proteins modulate chromatin structure under stress conditions.	Neurodegenerative diseases, cancer	^159^, ^160^
Hypoxia‐induced chromatin states	HIF‐targeted gene loci	Differential methylation of HIF‐responsive elements modulates gene expression.	Cancer, ischemic diseases	^161^
Reversible DNA modifications	TET enzymes, hydroxymethylation	Reactivates silenced genes in response to environmental cues.	Cancer, metabolic reprogramming	^162^
Histone acylation	p300, HDAC inhibitors	Regulates gene expression in response to metabolic shifts.	Cancer, heart disease	^162^

Abbreviations: APP, amyloid precursor protein; BRD4, bromodomain‐containing protein 4; circRNAs, circular RNAs; HDACs, histone deacetylases; HIF‐1α, hypoxia‐inducible factor 1 alpha; IKKβ, IκB kinase beta (inhibitor of nuclear factor kappa‐B kinase subunit beta); lncRNAs, long noncoding RNAs; miR‐210, microRNA‐210; miR‐23b‐27b, microRNA‐23b‐27b cluster; NF‐κB, nuclear factor kappa B; OTUB1, OTU domain‐containing ubiquitin aldehyde‐binding proteins 1; p53, tumor protein p53; SWI/SNF complex, switch/sucrose nonfermentable complex (a chromatin remodeling complex); TET enzymes, ten–eleven translocation enzymes.

Prenatal hypoxia influences gene expression through epigenetic modifications, including histone acetylation and DNA methylation, which alter fetal development and predispose individuals to aging‐related pathologies. In a lamb model of high‐altitude prenatal hypoxia, reduced histone acetylation and DNA methylation in fetal pulmonary arteries were linked to pulmonary arterial remodeling and hypertension in newborns.^141^ Similarly, maternal hypoxia decreased hepatic G6Pase expression in male offspring, associated with increased histone methylation around the G6Pase promoter.^142^ Hypoxia also modulates neurodevelopmental pathways, as seen in mid‐gestational neural precursor cells where HIF1α‐notch signaling promotes astrocyte differentiation through DNA demethylation of astrocytic genes, which may alter neuron–astrocyte ratios in the brain.^143^ Hypoxia‐induced hypermethylation of glucocorticoid receptor genes in the developing rat brain disrupts transcription factor binding and gene expression, impacting stress responses.^144^ Also, demethylation of the corticotropin‐releasing hormone gene (Crhr1) in the hypothalamus of male offspring exposed to intermittent hypoxia contributes to anxiety‐like behaviors in adulthood.^145^ These modifications can result in cognitive deficits and increased susceptibility to neurodegeneration, highlighting the significant role of hypoxia‐induced chromatin changes in brain development.

Hypoxia alters microRNA (miRNA) expression in neuronal cells, as seen in studies on rat cortical pericytes and hippocampus, linking altered miRNA profiles to cognitive dysfunction and neurodegenerative phenotypes.^146^, ^147^ miRNAs, such as miR‐210 and miR‐23b‐27b, are specifically regulated by hypoxia via HIF‐1α and other transcription factors, contributing to neuronal apoptosis and altered brain development.^148^, ^149^ Changes in miRNA expression are also associated with neurodegenerative disorders like Alzheimer's disease (AD), Parkinson's disease (PD), and Huntington's disease (HD), suggesting that hypoxia‐induced miRNA dysregulation in developing brains can predispose individuals to neurodegeneration later in life.^150^, ^151^, ^152^ Quantifying hypoxia‐regulated miRNAs in maternal blood may offer a noninvasive method to assess fetal hypoxia risk and related neurodevelopmental issues.^153^ In Alzheimer's models, hypoxia‐induced demethylation and reduced DNA methyltransferase 3b expression elevated levels of amyloid precursor protein (APP), β‐ and γ‐secretases, which are linked to learning and memory deficits in offspring, underscoring the long‐term impact of hypoxic epigenetic changes on neurodegeneration.^5^

## 3 DISEASES ASSOCIATED WITH HYPOXIA AND AGING

The impact of hypoxia on the aging process extends beyond cellular and molecular alterations, contributing to the pathogenesis of numerous age‐related diseases. CVDs, such as atherosclerosis, hypertension, and heart failure, are significantly influenced by hypoxia‐induced oxidative stress and endothelial dysfunction, which compromise vascular integrity and promote pathological remodeling.^121^, ^122^ Hypoxia also exacerbates neurodegenerative disorders like AD and PD by impairing mitochondrial function, increasing ROS production, and triggering neuroinflammation, all of which contribute to neuronal loss and cognitive decline.^125^, ^163^ Similarly, hypoxic conditions in adipose tissue have been linked to metabolic disorders, including type 2 diabetes and obesity, where inflammation and insulin resistance are driven by adipocyte dysfunction under low‐oxygen availability.^123^, ^124^

Pulmonary diseases, such as chronic COPD and idiopathic pulmonary fibrosis, are also closely associated with hypoxia, particularly in aging populations. Hypoxia promotes fibroblast activation and excessive extracellular matrix deposition, leading to progressive lung function decline.^104^, ^105^ Furthermore, hypoxia‐induced activation of HIF pathways has been implicated in cancer progression, as it enhances tumor angiogenesis, metabolic adaptation, and resistance to therapy, particularly in older individuals whose tissue environments are more prone to hypoxic stress.^164^, ^165^ The multifaceted impact of hypoxia on these diseases underscores the importance of targeting hypoxia‐induced pathways as a therapeutic strategy for mitigating the burden of age‐related pathologies.

### 3.1 Cardiovascular diseases

CVDs are among the leading causes of morbidity and mortality worldwide, and their incidence significantly increases with age. Hypoxia is a crucial factor in the pathogenesis of many cardiovascular conditions, including atherosclerosis, hypertension, and heart failure. Hypoxia‐induced oxidative stress and inflammation contribute to endothelial dysfunction, a key event in the development of atherosclerosis.^166^ Under hypoxic conditions, endothelial cells produce increased levels of ROS and proinflammatory cytokines, leading to vascular damage, plaque formation, and progression of atherosclerosis.^167^ Aging further exacerbates these effects by reducing endothelial cell function and enhancing oxidative stress, creating a vicious cycle that promotes cardiovascular pathology.

Hypertension, another common age‐related cardiovascular condition, is closely linked to hypoxia. Chronic hypoxia activates the renin–angiotensin–aldosterone system (RAAS), leading to increased blood pressure and vascular remodeling.^168^ Hypoxia‐induced activation of HIFs also promotes the expression of genes involved in vascular tone regulation, such as endothelin‐1 and nitric oxide synthase, contributing to hypertension.^169^ With aging, the capacity of the vascular system to respond to these hypoxic stimuli diminishes, resulting in sustained hypertension and increased risk of cardiovascular events.

Heart failure, a cardiovascular condition, is also exacerbated by hypoxia and aging. In heart failure, reduced cardiac output leads to tissue hypoxia, which triggers compensatory mechanisms, including HIF activation, to restore oxygen delivery.^170^ However, chronic hypoxia can lead to maladaptive cardiac remodeling, characterized by fibrosis, hypertrophy, and impaired contractility.^171^ Aging further impairs the heart's ability to adapt to hypoxic stress, contributing to the progression of heart failure and poor clinical outcomes.^172^ Targeting hypoxia‐related pathways may offer therapeutic benefits in managing age‐related CVDs.

Hypoxia has been linked to increased thrombosis, particularly in conditions like polycythemia vera and hypoxic cancer tissues.^173^ Studies demonstrated that HIF‐1α downregulates the antithrombotic factor protein S in liver cells, leading to increased thrombin and a prothrombotic state. This suggests that hypoxia‐driven HIF‐1α activity contributes to thrombotic risk by altering key antithrombotic pathways.

Acute mountain sickness (AMS) arises from complex physiological responses to hypoxia, including inflammation, vasogenic edema, and acidosis. Recent studies indicate that proinflammatory cytokines such as IL‐1β, IL‐6, and TNF‐α are elevated in individuals exposed to high altitudes, suggesting inflammation's role in AMS development.^99^, ^100^, ^101^, ^102^, ^103^ AMS‐susceptible individuals show lower anti‐inflammatory markers like IL‐10 and higher proinflammatory markers, highlighting a potential imbalance in inflammatory responses.^101^, ^174^ The disruption of the blood–brain barrier and vasogenic edema due to hypoxia‐driven inflammatory mediators may contribute to AMS symptoms, although the exact mechanisms remain to be fully elucidated.

High‐altitude cerebral edema (HACE) is a severe condition that often develops after AMS and involves hypoxia‐mediated cerebral vasodilation, loss of blood–brain barrier integrity, and increased intracranial pressure.^175^, ^176^ Proinflammatory cytokines and increased AQP4 activity in astrocytes, triggered by TLR4 and MAPK signaling, exacerbate astrocyte permeability and contribute to vasogenic edema.^99^, ^110^ Although inflammation likely plays a role in HACE development, further research is needed to clarify its exact contribution and how it interacts with hypoxic pathways.

High‐altitude pulmonary hypertension (HAPH) results from generalized hypoxic pulmonary vasoconstriction (HPV), leading to increased pulmonary artery pressures. Chronic hypoxia induces a proinflammatory microenvironment that drives vascular remodeling, marked by increased vessel muscularization and inflammatory cell recruitment.^177^, ^178^ High‐altitude pulmonary edema (HAPE) is primarily caused by exaggerated HPV, acute pulmonary hypertension, and increased capillary permeability, leading to alveolar fluid accumulation.^179^, ^180^, ^181^, ^182^ Inflammation appears to contribute to HAPE susceptibility, with HAPE‐prone individuals showing elevated baseline inflammation and reduced lung function compared to resistant individuals.^111^, ^112^ Pre‐existing pulmonary vascular remodeling and chronic inflammation may further exacerbate susceptibility, though direct mechanisms remain under investigation.^183^

### 3.2 Neurodegenerative disorders

Neurodegenerative disorders, including AD, PD, and HD, are characterized by the progressive loss of neurons and cognitive decline. Hypoxia is a known contributor to the pathogenesis of these disorders through mechanisms involving oxidative stress, inflammation, and mitochondrial dysfunction.^184^, ^185^, ^186^ In AD, hypoxia exacerbates amyloid beta (Aβ) aggregation and tau hyperphosphorylation, two hallmark features of the disease, by increasing ROS production and activating inflammatory pathways.^107^, ^108^, ^109^ Studies show that chronic hypoxia induces blood–brain barrier disruption, further promoting neuroinflammation and neuronal damage in aging brains. Hypoxia upregulates β‐ and γ‐secretases, key enzymes in Aβ production, and reduces neprilysin (NEP), an enzyme involved in Aβ degradation.^187^, ^188^ Animal studies have shown that hypoxic conditions enhance tau phosphorylation, disrupt APP processing, and impair memory, indicating that hypoxia accelerates AD progression through multiple molecular pathways.^108^, ^109^, ^189^ Antenatal hypoxia has been linked to early onset AD‐related pathology, particularly in genetically susceptible models such as 5 × Familial Alzheimer's Disease (FAD) mice. Hypoxia during fetal development exacerbates cognitive decline and neuroinflammation, increasing the vulnerability of the brain to AD‐related damage later in life.^190^ This highlights a potential developmental origin of aging‐related neurodegenerative disorders influenced by hypoxic conditions. Hypoxia and ischemia are known to worsen cerebrovascular dysfunctions seen in AD, such as reduced cerebral blood flow and capillary amyloid angiopathy. These hypoxic conditions elevate β‐ and γ‐secretase activities, increasing Aβ deposition and contributing to AD pathology.^191^ HIF‐1α plays a critical role in this process by upregulating secretase activities and interacting with key components of the Aβ pathway, reinforcing the link between hypoxia, vascular dysfunction, and AD progression.

In PD, hypoxia contributes to dopaminergic neuron loss in the substantia nigra, a key pathological feature of the disease. Hypoxic conditions have been shown to impair mitochondrial function and increase oxidative stress in dopaminergic neurons, leading to their degeneration.^192^ Furthermore, hypoxia activates HIF‐1α, which modulates the expression of genes involved in apoptosis and autophagy, processes implicated in PD progression. As the brain's capacity to cope with hypoxic stress declines with age, the risk of developing PD increases, highlighting the importance of understanding hypoxia's role in neurodegeneration.^193^ Hypoxia also plays a role in the progression of HD, a genetic neurodegenerative disorder characterized by the accumulation of mutant huntingtin protein. Research indicates that hypoxia can exacerbate mitochondrial dysfunction and enhance the aggregation of mutant huntingtin, leading to neuronal death. Moreover, hypoxia‐induced oxidative stress and inflammation contribute to the loss of striatal neurons, a hallmark of HD pathology.^194^, ^195^, ^196^ These findings suggest that hypoxia is a common pathogenic factor in various neurodegenerative diseases, particularly in the context of aging.

### 3.3 Cancer

Cancer is a complex and multifaceted disease that becomes increasingly prevalent with age, partly due to the accumulation of cellular damage and the diminished ability to respond to stressors like hypoxia. Hypoxia is a critical driver of tumorigenesis, especially in aging populations, as it promotes genetic instability, metabolic reprogramming, and resistance to cell death.^197^ Hypoxic tumor microenvironments, characterized by low‐oxygen levels, stabilize HIFs, which induce the expression of genes involved in angiogenesis, cell survival, and invasion, contributing to cancer progression.^198^

One of the key ways that hypoxia contributes to tumor progression is by promoting angiogenesis—the formation of new blood vessels essential for tumor growth and metastasis. HIF‐1α upregulates vascular endothelial growth factor (VEGF), a crucial mediator of angiogenesis, thereby facilitating tumor expansion.^113^, ^199^ Aging tissues are more susceptible to hypoxia‐induced DNA damage and cellular transformation, increasing the risk of cancer development. Additionally, the ability to respond to hypoxic stress diminishes with age, leading to more aggressive tumor phenotypes and poorer clinical outcomes.^10^, ^200^

Hypoxia also induces significant metabolic reprogramming in cancer cells. Under hypoxic conditions, cancer cells shift their energy production from oxidative phosphorylation to glycolysis—a phenomenon known as the “Warburg effect”. This metabolic shift provides a growth advantage to tumor cells, allowing them to thrive even in low‐oxygen environments.^201^, ^202^ Moreover, hypoxic conditions enhance the resistance of tumor cells to therapies such as chemotherapy and radiation. These cells exhibit altered metabolism, increased expression of drug efflux pumps, and enhanced DNA repair capacity, making them less responsive to conventional treatments. Hypoxia also supports the maintenance of cancer stem cells, which are implicated in tumor recurrence and metastasis, posing a significant challenge in cancer treatment, particularly in older patients with reduced physiological reserve.^203^

The interplay between hypoxia and inflammation further complicates cancer progression, particularly through the activation of NF‐κB signaling. In colorectal cancer (CRC), hypoxia‐induced NF‐κB activation drives tumor growth, angiogenesis, and metastasis.^106^ HIF‐1α and HIF‐2α have different roles in CRC, with HIF‐1α driving cancer development and HIF‐2α acting as a tumor suppressor.^204^ However, in colon cancer, HIF‐2α plays a crucial role in promoting cancer progression by activating inflammatory pathways. Specifically, it increases the production of proinflammatory mediators like TNF‐α, which is a key contributor to cancer development.^205^ Studies have demonstrated that anti‐inflammatory drugs such as nimesulide, which inhibit HIF‐2α, can significantly reduce tumor growth.^205^ The crosstalk between NF‐κB and Wnt/β‐catenin underscores the complex regulatory networks at play and highlights the need for targeted therapeutic strategies.

Similarly, in breast and lung cancers, NF‐κB and HIF signaling are pivotal in driving disease progression. In breast cancer, NF‐κB activation, often triggered by hypoxic and inflammatory stimuli, promotes epithelial–mesenchymal transition (EMT) and metastasis.^155^ In non–small‐cell lung carcinoma (NSCLC), NF‐κB activation contributes to resistance against tyrosine kinase inhibitors, underscoring its critical role in cancer survival and positioning it as a potential therapeutic target.^206^ The involvement of JmjC enzymes, such as KDM4 family members, further demonstrates the intricate relationship between inflammation, hypoxia, and cancer progression in these tumors.

Intra tumoral hypoxia significantly influences the immune landscape of tumors. In NSCLC, loss of HIF‐2α increases tumor burden and immune cell infiltration, particularly of granulocytic cells, indicating that HIF‐2α can exert antitumor effects by modulating immune responses.^207^ In contrast, the loss of HIF‐1α does not impact NSCLC progression, highlighting the distinct functional roles of HIF isoforms in cancer biology. In pancreatic ductal adenocarcinoma (PDAC), HIF‐1α and HIF‐2α also exhibit opposing effects on tumor progression: HIF‐1α deletion enhances tumor proliferation and immune infiltration, whereas loss of HIF‐2α reduces the progression of precursor lesions, further underscoring the nuanced roles of these factors in cancer.^208^, ^209^

### 3.4 Metabolic disorders

Metabolic disorders, including type 2 diabetes, obesity, and metabolic syndrome, are closely associated with aging and are exacerbated by hypoxia. Hypoxia affects metabolic homeostasis by altering glucose metabolism, lipid metabolism, and insulin signaling.^210^ Under hypoxic conditions, HIF‐1α activation promotes glycolysis and suppresses oxidative phosphorylation, leading to hyperglycemia and insulin resistance, key features of diabetes.^119^, ^211^, ^212^, ^213^ Additionally, hypoxia‐induced inflammation and oxidative stress contribute to pancreatic beta‐cell dysfunction, further impairing glucose homeostasis.^114^, ^115^

Obesity, a major risk factor for metabolic disorders, is also linked to hypoxia. Adipose tissue hypoxia in obese individuals leads to increased inflammation, adipocyte dysfunction, and insulin resistance.^124^, ^214^, ^215^ Hypoxia in adipose tissue promotes macrophage infiltration and the production of proinflammatory cytokines, which exacerbate metabolic dysregulation.^123^, ^213^ Aging further compounds these effects by reducing adipose tissue oxygenation and enhancing systemic inflammation, increasing the risk of metabolic diseases. Hypoxia affects lipid metabolism by regulating key enzymes involved in lipogenesis and lipolysis. HIF‐1α activation under hypoxic conditions promotes lipogenesis while inhibiting lipolysis, leading to lipid accumulation and dyslipidemia, which are common in aging individuals. Hypoxia‐induced dyslipidemia contributes to the development of CVDs and other metabolic complications, further linking hypoxia to age‐related metabolic disorders.^216^, ^217^

### 3.5 Pulmonary diseases

Pulmonary diseases, including COPD and pulmonary fibrosis, are common in aging populations and are significantly influenced by hypoxia. Hypoxia plays a central role in the pathogenesis of these conditions by promoting inflammation, fibrosis, and tissue remodeling.^218^, ^219^ In COPD, hypoxia‐induced oxidative stress and inflammation contribute to airway remodeling, alveolar destruction, and impaired gas exchange.^219^, ^220^, ^221^, ^222^ Aging further exacerbates these effects by reducing lung function and increasing susceptibility to hypoxia‐induced damage.^223^ In COPD, acute hypoxic exposure triggers both inflammatory and coagulation responses, marked by increased levels of thrombin–antithrombin complexes (TAT), D‐dimers, and von Willebrand factor (VWF) antigen, along with elevated inflammatory mediators.^224^

Hypoxia promotes fibroblast activation, extracellular matrix deposition, and tissue remodeling, leading to the development and progression of pulmonary fibrosis. Aging is a significant risk factor for pulmonary fibrosis, as the capacity for tissue repair and regeneration declines with age. Hypoxia‐induced activation of HIF‐1α and other profibrotic pathways further accelerates disease progression in aging individuals.

### 3.6 Prenatal hypoxia and aging

Prenatal hypoxia, resulting from complications during pregnancy, is a critical factor influencing fetal brain development, leading to long‐term consequences in postnatal life. Hypoxic conditions during gestation have been linked to an increased risk of various central nervous system (CNS) disorders, including autism, schizophrenia, and neurodegenerative diseases such as PD and AD.^225^ Prenatal hypoxia alters gene expression and protein metabolism, notably affecting key proteins like acetylcholinesterase and APP, which are crucial for brain function.^226^ The disruption of these pathways can lead to early cognitive dysfunctions and predispose individuals to neurodegenerative processes. Additionally, hypoxia impairs the activity of Aβ‐degrading enzymes, resulting in toxic Aβ accumulation and neuronal damage.^227^

Prenatal hypoxia also affects the body's response to hypoxia later in life by altering catecholaminergic components of the chemoafferent pathway, impairing respiratory behavior, and increasing oxidative stress.^228^ These findings highlight the importance of addressing prenatal hypoxia as a modifiable risk factor for aging‐related cognitive decline and neurodegeneration.

### 3.7 Obstructive sleep apnea‐induced intermittent hypoxia and aging

Obstructive sleep apnea (OSA) is characterized by repeated episodes of upper airway obstruction leading to intermittent hypoxia. OSA‐induced cyclical hypoxemia–reoxygenation activates chemoreceptors, causing sympathetic overactivation, increased blood pressure, and chronic intermittent hypoxia (CIH).^229^ These hypoxic episodes contribute to molecular and cellular impairments that accelerate the progression of various diseases and aging.^230^ The OSA is associated with pulmonary hypertension, as demonstrated in highland populations, where sleep apnea and hypoxemia were linked to increased pulmonary artery pressure.^231^, ^232^ The recurrent hypoxic stress triggers inflammatory responses, sympathetic activation, and vascular damage, contributing to conditions like atherosclerosis and further aggravating pulmonary hypertension.^12^, ^13^, ^14^, ^15^, ^16^, ^17^, ^18^, ^19^, ^20^, ^21^, ^22^, ^23^, ^24^, ^25^, ^26^, ^27^, ^28^, ^29^, ^30^, ^31^, ^32^, ^33^, ^34^, ^35^, ^36^, ^37^, ^38^, ^39^, ^40^, ^41^, ^42^, ^43^, ^44^, ^45^, ^46^, ^47^, ^48^, ^49^, ^50^, ^51^, ^52^, ^53^, ^54^, ^55^, ^56^, ^57^, ^58^, ^59^, ^60^, ^61^, ^62^, ^63^, ^64^, ^65^, ^66^, ^67^, ^68^, ^69^, ^70^, ^71^, ^72^, ^73^, ^74^, ^75^, ^76^, ^77^, ^78^, ^79^, ^80^, ^81^, ^82^, ^83^, ^84^, ^85^, ^86^, ^87^, ^88^, ^89^, ^90^, ^91^, ^92^, ^93^, ^94^, ^95^, ^96^, ^97^, ^98^, ^99^, ^100^, ^101^, ^102^, ^103^, ^104^, ^105^, ^106^, ^107^, ^108^, ^109^, ^110^, ^111^, ^112^, ^113^, ^114^ This suggests a complex interplay between OSA, intermittent hypoxia, and cardiovascular risk, underscoring the need for targeted therapeutic interventions to mitigate the adverse effects of hypoxia‐induced aging.

The OSA is more common in aging and is associated with comorbidities such as male sexual dysfunction. Oxidative stress, which increases with both age and CIH, is a proposed mechanism linking sleep apnea to sexual dysfunction.^233^ Studies in animal models of CIH showed that middle‐aged male rats experienced elevated oxidative stress, reduced testosterone, and increased sexual dysfunction. Notably, CIH had more profound effects on younger intact males, suggesting that hypoxia mimics aging‐related dysfunction in sexual behavior and oxidative stress.

## 4 THERAPEUTIC TARGETS AND INTERVENTIONS

**FIGURE 3:**
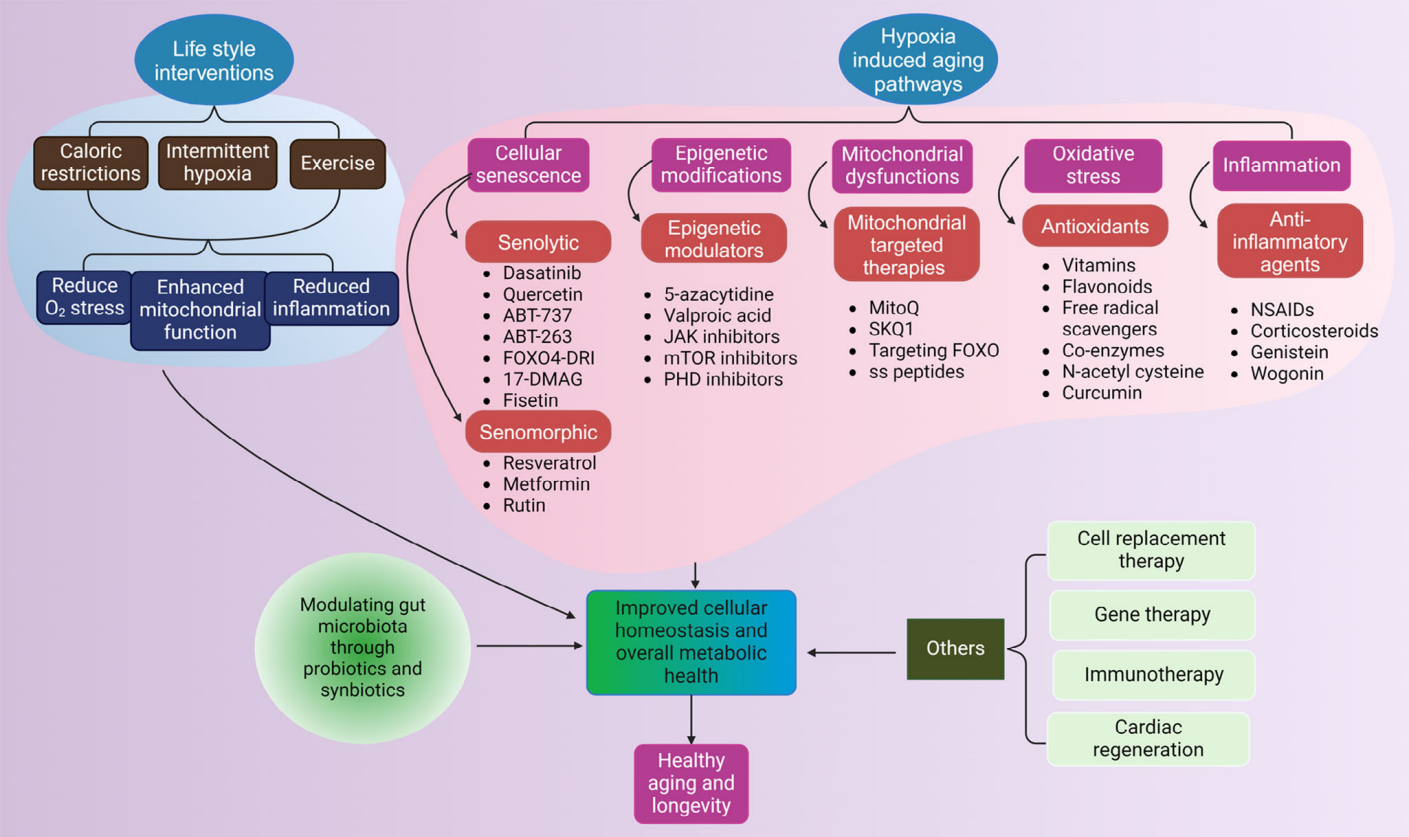
Overview of the therapeutic targets and lifestyle interventions for mitigating hypoxia‐induced aging. Various interventions and therapeutic strategies are outlined, which are aimed at alleviating the effects of hypoxia‐induced aging. Key interventions include lifestyle modifications such as caloric restriction, intermittent hypoxia, and exercise, which enhance mitochondrial function, reduce oxidative stress, and decrease inflammation. Therapeutic targets include cellular senescence modifiers (e.g., senolytics like dasatinib and quercetin), antioxidants (e.g., vitamins, flavonoids, N‐acetyl cysteine), and anti‐inflammatory agents (e.g., nonsteroidal anti‐inflammatory drugs [NSAIDs], corticosteroids). Additional approaches include epigenetic modulators (e.g., 5‐azacytidine, mechanistic target of rapamycin [mTOR] inhibitors), mitochondrial‐targeted therapies (e.g., mitoquinone [MitoQ], SKQ1), and advanced treatments like cell replacement therapy, gene therapy, and immunotherapy. These approaches aim to improve mitochondrial function, reduce inflammation, and promote healthy aging and longevity.

Given the critical role of hypoxia in aging and related diseases, therapeutic strategies targeting hypoxia‐responsive pathways are increasingly under investigation. Modulation of HIF pathways, either by inhibition or controlled activation, offers a promising approach to restore cellular homeostasis and enhance resistance to hypoxic stress (Figure 3). Pharmacological interventions targeting HIFs, alongside antioxidants and mitochondrial‐directed therapies like mitoquinone (MitoQ), demonstrate potential in mitigating oxidative stress and improving mitochondrial function. Moreover, senolytic agents, anti‐inflammatory compounds, and epigenetic modulators are being explored to address hypoxia‐induced cellular damage. Complementary lifestyle interventions, including exercise and caloric restriction (CR), may further optimize these therapeutic strategies, paving the way for personalized approaches to promote healthy aging (Table 4).

**TABLE 4:** Therapeutic targets for mitigating hypoxia‐induced aging and diseases.

Therapeutic target	Mechanism of action	Potential benefits	Relevant diseases	References
HIF modulators	Regulates hypoxia‐responsive pathways	Reduces hypoxia‐induced inflammation and tumor growth.	Cancer, cardiovascular diseases	^234^, ^235^, ^236^
Antioxidants	Scavenges ROS, reduces oxidative stress	Protects against cellular damage and aging.	Neurodegeneration, COPD	^237^, ^238^
Anti‐inflammatory agents	Inhibits NF‐κB and TNF‐α pathways	Lowers inflammation, slows disease progression.	CRC, Alzheimer's disease	^239^, ^240^
Lifestyle interventions	Intermittent hypoxia training, caloric restriction	Enhances stress resilience, improves mitochondrial function.	Aging, metabolic dysfunctions	^241^, ^242^
SIRT1 activators	Enhances autophagy and cellular stress response	Extends lifespan and protects against metabolic disorders.	Aging, diabetes	^243^
PHD inhibitors	Stabilizes HIFs, modulates oxygen sensing	Promotes angiogenesis and tissue repair in ischemic conditions.	Stroke, myocardial infarction	^244^
Epigenetic drugs	DNMT inhibitors, HDAC inhibitors	Reverses harmful epigenetic changes, restores normal gene function.	Cancer, neurodegeneration	^245^
Mitochondrial‐targeted therapies	MitoQ, SS peptides	Enhances mitochondrial function, reduces ROS production.	Cardiovascular diseases, aging	^246^, ^247^
Senolytics	Clears senescent cells, reduces SASP	Delays aging‐related tissue dysfunction.	Aging, osteoarthritis	^248^, ^249^, ^250^
Immunomodulatory agents	IL‐10, TGF‐β blockers	Modulates immune response, reduces chronic inflammation.	Brain damage, autoimmune diseases, aging	^251^
Stem cell therapy	Replaces damaged cells, rejuvenates tissues	Regenerative potential in aging and degenerative diseases.	Parkinson's, spinal cord injury	^252^, ^253^
Telomerase activators	Lengthens telomeres, delays senescence	Potential to extend lifespan and improve cell viability.	Aging, cardiovascular diseases	^254^
Redox modulators	Nrf2 activators	Enhances antioxidant defense, reduces oxidative damage.	Aging, metabolic syndrome	^70^
Metformin	AMPK activation, reduces insulin resistance	Extends healthspan, protects against aging‐related diseases.	Diabetes, aging	^255^

Abbreviations: AMPK, AMP‐activated protein kinase; COPD, chronic obstructive pulmonary disease; CRC, colorectal cancer; DNMT, DNA methyltransferase; HDAC, histone deacetylase; HIF, hypoxia‐inducible factor; IL‐10, interleukin‐10; MitoQ, mitoquinone; NF‐κB, nuclear factor kappa B; Nrf2, nuclear factor erythroid 2‐related factor 2; PHD, prolyl hydroxylase; ROS, reactive oxygen species; SASP, senescence‐associated secretory phenotype; SS peptides, Szeto–Schiller peptides; TGF‐β, transforming growth factor beta; TNF‐α, tumor necrosis factor alpha.

### 4.1 Targeting hypoxia‐inducible factors

HIFs, particularly HIF‐1α, are key regulators of the cellular response to hypoxia and represent promising therapeutic targets for aging and age‐related diseases. Pharmacological modulation of HIF pathways has the potential to enhance adaptive responses to hypoxia, improve metabolic homeostasis, and reduce inflammation.^256^ While HIF inhibition has shown promise in preclinical models, its therapeutic potential in aging remains controversial. Chronic HIF activation has been associated with increased tumorigenesis and other pathological conditions, raising concerns about the long‐term safety of HIF‐targeted therapies.^257^ However, recent studies suggest that selective modulation of HIF pathways, such as transient HIF activation or targeting specific HIF isoforms, may offer a safer and more effective approach for promoting healthy aging.^234^, ^235^, ^236^

In addition to HIF inhibitors, other strategies for targeting HIF pathways include the use of small molecules, peptides, and gene therapy approaches to modulate HIF activity.^258^ These approaches have shown promise in preclinical models of aging and age‐related diseases, but further research is needed to determine their efficacy and safety in humans. Overall, targeting HIFs represents a promising avenue for therapeutic intervention in aging, but careful consideration of the risks and benefits is essential.

### 4.2 Antioxidants and mitochondrial therapies

Given the central role of oxidative stress in aging and age‐related diseases, antioxidants have been extensively studied as potential therapeutic agents. Antioxidants, such as vitamins C and E, coenzyme Q10, and N‐acetylcysteine, aim to neutralize ROS and reduce oxidative damage.^237^, ^238^ However, the efficacy of antioxidant therapy in promoting healthy aging and preventing age‐related diseases remains uncertain, with some studies showing limited benefits or even potential harm. This has led to a shift in focus toward more targeted approaches, such as mitochondrial‐targeted antioxidants and therapies that enhance endogenous antioxidant defenses.^259^ These antioxidants, such as MitoQ and SkQ1, are designed to accumulate selectively within mitochondria, where they can more effectively neutralize ROS and reduce mitochondrial damage.^246^, ^247^ Preclinical studies have shown that these compounds can improve mitochondrial function, reduce oxidative stress, and extend lifespan in animal models.^246^, ^260^, ^261^ Clinical trials are currently underway to evaluate the efficacy of mitochondrial‐targeted antioxidants in human aging and age‐related diseases.^262^

In addition to antioxidant therapies, other mitochondrial‐targeted interventions, such as agents that enhance mitochondrial biogenesis, mitophagy, and metabolic flexibility like FOXO, are being explored for their potential to promote healthy aging.^263^, ^264^ These approaches aim to restore mitochondrial function and improve cellular energy metabolism, which may help mitigate the effects of hypoxia and oxidative stress on aging. However, further research is needed to identify the most effective strategies for targeting mitochondrial dysfunction in aging.

### 4.3 Anti‐inflammatory and senolytic agents

Chronic inflammation, or “inflammaging” is a key driver of aging and age‐related diseases, making anti‐inflammatory agents a promising therapeutic target. Nonsteroidal anti‐inflammatory drugs (NSAIDs), corticosteroids, and other anti‐inflammatory agents have been studied for their potential to reduce inflammation and improve health outcomes in aging populations.^239^, ^240^ However, these agents have significant side effects and may not be suitable for long‐term use in the elderly.^265^

Senolytic agents, which selectively eliminate senescent cells, have emerged as a novel therapeutic strategy for promoting healthy aging. Senescent cells accumulate with age and secrete proinflammatory factors, contributing to chronic inflammation and tissue dysfunction.^266^ Senolytic drugs, such as dasatinib and quercetin, have shown promise in preclinical models of aging by reducing senescent cell burden, improving tissue function, and extending lifespan.^248^, ^249^, ^250^ Clinical trials are currently underway to evaluate the safety and efficacy of senolytic agents in humans.^267^, ^268^, ^269^ Several other senolytic drugs such as ABT‐737, ABT‐263, FOXO4‐DRI (D‐retro inverso), and the HSP90 inhibitor 17‐DMAG primarily exert their senolytic effects by inducing apoptosis and mitochondrial dysfunction. However, their interactions with epigenetic mechanisms require further exploration.^270^, ^271^, ^272^, ^273^

In addition to senolytics, senomorphic agents such as rutin that modulate the SASP without eliminating senescent cells are being explored as a potential therapeutic approach.^274^ These agents aim to reduce the proinflammatory and profibrotic effects of senescent cells, potentially slowing the progression of age‐related diseases and improving healthspan.^275^ Further research is needed to determine the optimal strategies for targeting inflammation and senescence in aging.

### 4.4 Epigenetic modulators

Epigenetic changes, including DNA methylation, histone modifications, and noncoding RNA regulation, play a crucial role in aging and age‐related diseases. Epigenetic modulators, such as DNA methyltransferase inhibitors (e.g., 5‐azacytidine) and histone deacetylase (HDAC) inhibitors (e.g., valproic acid), have been studied for their potential to reverse age‐associated epigenetic changes and promote healthy aging.^276^ Targeting specific epigenetic marks associated with aging, such as hypermethylation of tumor suppressor genes or hypoacetylation of histones, may offer therapeutic benefits in age‐related diseases. HDACs, which act as corepressors in conjunction with histone acetyltransferases (HATs), play a significant role in longevity. Among them, sirtuins, a class of HDACs, are particularly important for maintaining genome stability. SIRT6, a member of this class, functions as an NAD^+^‐dependent H3K9 deacetylase, influencing telomeric chromatin.^277^ Overexpression of SIRT6 has been linked to increased longevity in both rat and human nucleus pulposus cells by inhibiting cellular senescence.^243^

Emerging research suggests that lifestyle interventions, such as diet, exercise, and environmental exposures, may also influence the epigenome and promote healthy aging. Understanding the role of epigenetics in aging may reveal novel targets for therapeutic intervention and help identify individuals who are most likely to benefit from specific treatments. Future research should focus on identifying safe and effective strategies for modulating the epigenome to promote healthy aging and prevent age‐related diseases.

### 4.5 Lifestyle and environmental interventions

Lifestyle and environmental interventions, such as exercise, CR, and exposure to intermittent hypoxia, have been shown to promote healthy aging and reduce the risk of age‐related diseases. Exercise is a well‐established intervention that can significantly impact epigenetic modifications, influencing gene expression and contributing to various health benefits. Physical activity has been shown to remodel DNA methylation patterns on the promoters of key genes in skeletal muscle,^278^ and histone modifications may also be affected through the inhibition of HDACs, thus altering gene expression profiles.^245^ Additionally, exercise can regulate the expression of miRNAs, which play crucial roles in mediating these beneficial effects.^241^ For example, after just 8 weeks of voluntary resistance training, aged mice demonstrate an epigenetic rejuvenation equivalent to about 8 weeks of younger muscle tissue, along with a modest extension in lifespan.^279^ Similarly, voluntary wheel running in aged mice enhances neurogenesis, improves learning abilities, and mitigates age‐related synaptic abnormalities.^280^ These rejuvenating effects are not limited to animal models; they are also evident in humans. Physically active elderly individuals show a significantly different transcriptional profile compared to their sedentary counterparts, and endurance exercise has been found to improve muscle function in older adults.^281^

CR, a dietary strategy that lowers calorie intake without causing malnutrition, has been associated with increased lifespan and enhanced health across various animal studies. By reducing calorie consumption by 10%–40%, CR has been shown to significantly extend the lifespan of rodents.^242^ This intervention also offers several health benefits, including improved vascular endothelial function, enhanced aerobic capacity of skeletal muscles, and a reduction in muscle fiber loss and motor neuron turnover.^282^, ^283^, ^284^, ^285^

Intermittent hypoxia, a practice that involves short‐term exposure to low‐oxygen levels, has been studied for its potential to mimic the benefits of exercise and promote healthy aging.^286^ Intermittent hypoxia training has been shown to improve neuronal function, enhance mitochondrial biogenesis, and reduce oxidative stress in animal models.^287^, ^288^, ^289^ Recently, a study conducted by researchers at Massachusetts General Hospital explored the effects of oxygen restriction on longevity in mice.^290^ The study revealed significant findings, demonstrating that oxygen restriction led to notable improvements in lifespan and delayed neurological decline in mice. Mice exposed to mild hypoxia exhibited an impressive 50% increase in lifespan compared to control animals maintained under normoxic conditions. Additionally, hypoxia‐exposed mice showed delayed onset and progression of neurological decline, suggesting a protective effect against age‐related neurodegenerative processes. However, the long‐term safety and efficacy of this intervention in humans remain uncertain.

Overall, dietary modifications and physical exercise remain the most widely endorsed approaches for addressing aging and related diseases, largely due to their proven efficacy and safety. While recent developments in pharmacological treatments, such as geroprotectors, have demonstrated potential benefits for various age‐related conditions, concerns about their safety and variable outcomes underscore the need for additional clinical research to establish their reliability.

## 5 CHALLENGES AND FUTURE DIRECTIONS

**FIGURE 4:**
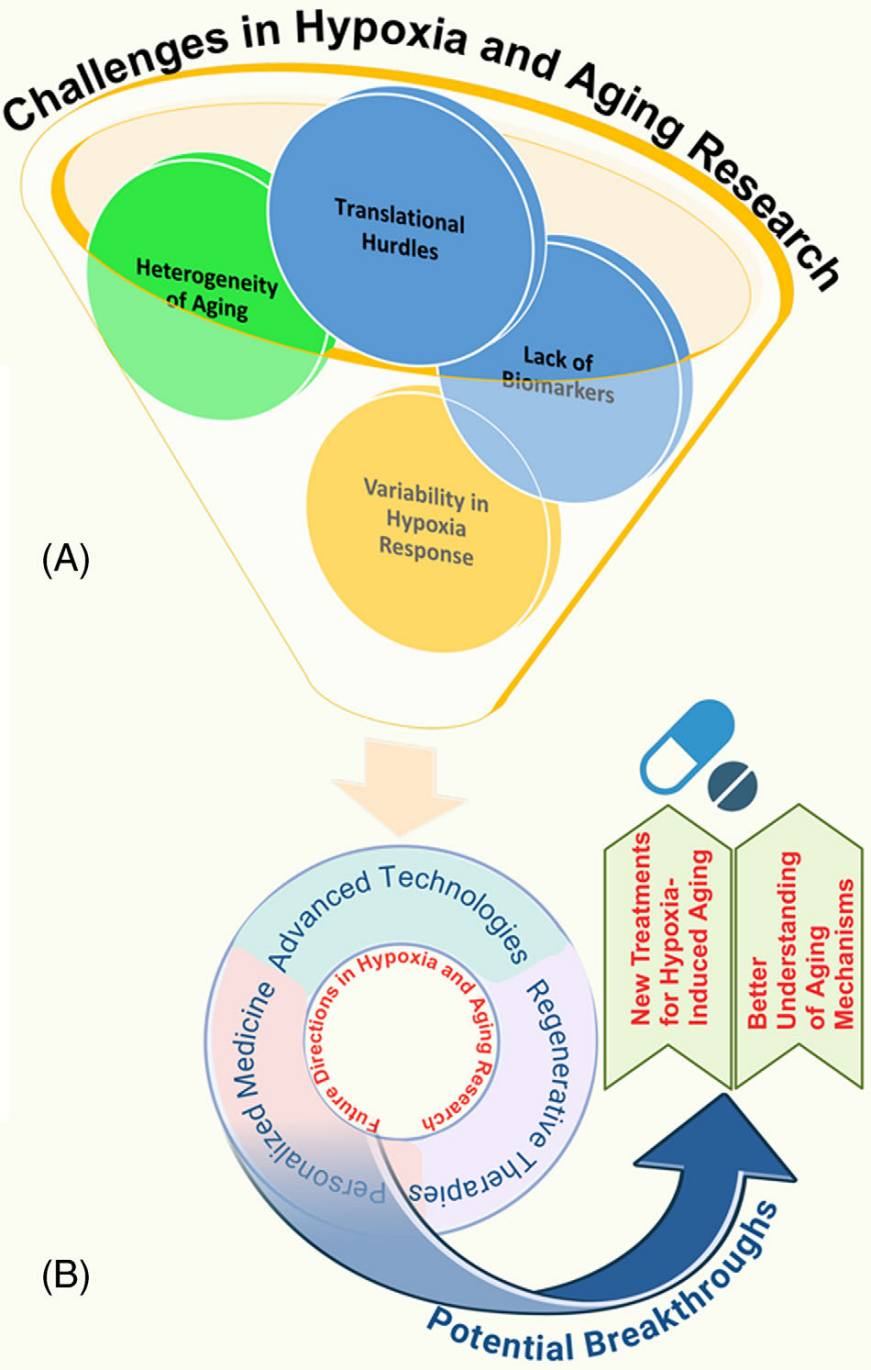
Infographic of challenges and future directions in hypoxia and aging research. The challenges and future directions in the field of hypoxia and aging research are highlighted. Challenges in hypoxia and aging research include lack of biomarkers, variability in hypoxia response, heterogeneity of aging, and translational hurdles. Emphasized areas for future research include the development of advanced technologies, regenerative therapies, and personalized medicine to better understand the molecular mechanisms driving aging under hypoxic conditions. This could lead to potential breakthroughs such as new treatments for hypoxia‐induced aging and better understanding of aging mechanisms.

While the therapeutic potential of targeting hypoxia‐induced pathways in aging is promising, significant challenges remain in translating these findings into clinical practice (Figure 4A). A critical issue is the lack of reliable biomarkers that can accurately measure the impact of hypoxia on aging and monitor the effectiveness of therapeutic interventions.^291^ Existing biomarkers do not fully capture the multifaceted effects of hypoxia on cellular and systemic aging processes, making it difficult to assess the efficacy and safety of new treatments. Developing robust biomarkers is essential for guiding the clinical application of hypoxia‐targeted therapies and advancing personalized treatment strategies.

The heterogeneity of aging presents another significant challenge, as aging is influenced by genetic, environmental, and lifestyle factors that vary widely among individuals.^292^, ^293^, ^294^ This variability complicates the identification of universal mechanisms and therapeutic targets, as the impact of hypoxia on aging can differ significantly between tissues and individuals. Additionally, variability in hypoxia response, such as differences in HIF signaling and mitochondrial resilience, further complicates the development of broadly effective treatments.^295^ These differences underscore the importance of personalized approaches that consider individual genetic and epigenetic profiles to tailor interventions effectively.

Translational hurdles also impede the clinical application of hypoxia‐targeted therapies.^296^ The safety and long‐term effects of pharmacological agents, such as HIF modulators, antioxidants, and senolytics, need thorough evaluation to minimize potential adverse outcomes, including the risk of cancer or other unintended consequences.^297^ Additionally, the multifactorial nature of aging and its associated diseases makes it challenging to design targeted therapies without off‐target effects. Innovative clinical trial designs and personalized approaches are needed to address these challenges and evaluate new therapies effectively.

However, hypoxia does not always exert detrimental effects. In certain contexts, controlled hypoxia can have beneficial outcomes, such as enhancing tissue repair, stimulating angiogenesis, and promoting metabolic adaptation. This paradox, where hypoxia can both accelerate aging through mechanisms like oxidative stress, inflammation, and cellular senescence, and simultaneously exert protective effects under certain conditions, presents a unique challenge. Future strategies must aim to balance these opposing effects by identifying when and how hypoxia can be harnessed for its protective properties without exacerbating aging‐related damage. Future research should also explore the beneficial roles of controlled hypoxia in aging and its potential therapeutic applications. For example, intermittent hypoxia or hypoxia preconditioning has shown promise in improving resilience to stress and enhancing tissue regeneration in aging populations. Integrating these beneficial aspects of hypoxia into therapeutic strategies could open new avenues for promoting healthy aging while mitigating its harmful effects.

Future research in hypoxia and aging should leverage advanced technologies, such as single‐cell sequencing, CRISPR gene editing, and advanced imaging techniques, to gain deeper insights into cellular and molecular responses to hypoxia in aging tissues (Figure 4B). These technologies provide unprecedented opportunities to dissect the complex interactions between hypoxia and aging at the single‐cell level, enabling the identification of new therapeutic targets and potential biomarkers. Personalized medicine approaches, incorporating genomic, transcriptomic, and epigenomic data, can further refine interventions to match individual patient profiles, optimizing treatment outcomes and minimizing side effects.^298^, ^299^

Regenerative therapies and stem cell research also hold significant promise for addressing the effects of hypoxia on aging. Hypoxia influences stem cell function and differentiation, and understanding these effects could lead to new strategies for enhancing tissue regeneration and repair in aging individuals.^300^ Combining hypoxia‐modulating agents with regenerative approaches may improve the efficacy of stem cell‐based treatments, offering innovative solutions for combating age‐related decline.^301^ By integrating advanced technologies, personalized medicine, and regenerative therapies, future research has the potential to develop new treatments for hypoxia‐induced aging and deepen our understanding of aging mechanisms, ultimately improving healthspan and quality of life for aging populations.

## 6 CONCLUSION

The complex interplay between hypoxia and aging is mediated by a network of molecular mechanisms, including oxidative stress, inflammation, mitochondrial dysfunction, cellular senescence, and epigenetic modifications. These interconnected pathways contribute to the development and progression of various age‐related diseases, such as cardiovascular conditions, neurodegenerative disorders, cancer, metabolic syndromes, and pulmonary diseases. Understanding these mechanisms is essential for identifying potential therapeutic targets and developing strategies to promote healthy aging and mitigate the detrimental effects of hypoxia on aging processes.

Therapeutic interventions targeting hypoxia‐related pathways, such as HIF modulation, antioxidants, anti‐inflammatory agents, senolytics, epigenetic modulators, and lifestyle modifications, hold promise for improving health outcomes in aging populations. However, challenges in translating these findings into clinical practice include the need for reliable biomarkers, personalized approaches, and a deeper understanding of the long‐term effects of these interventions. Current research is limited by the absence of biomarkers that accurately reflect hypoxia's impact on aging processes at cellular, tissue, and systemic levels.^291^ Advances in “omics” technologies, such as proteomics and metabolomics, may help identify novel biomarkers that guide the development and assessment of these interventions.^297^ Given the heterogeneity in aging and individual differences in hypoxia response, personalized medicine approaches that consider genetic, epigenetic, and environmental factors are crucial. Precision medicine tools, such as genomic profiling, single‐cell RNA sequencing, and advanced imaging, can tailor interventions to individual needs, optimizing therapeutic outcomes and minimizing side effects.^298^, ^299^ Integrating machine learning and artificial intelligence to analyze large‐scale biological data could further accelerate discoveries in this field.^302^, ^303^ Nonetheless, emerging therapies targeting hypoxia‐induced pathways—such as HIF inhibitors, antioxidants, and senolytics—require rigorous preclinical and clinical evaluation to ensure their long‐term safety and efficacy.^304^ For instance, HIF inhibition might impair critical physiological responses needed for tissue repair, and chronic antioxidant use could disrupt normal redox signaling, highlighting the need to balance therapeutic benefits with potential risks.^305^

Regenerative medicine and stem cell therapies offer promising avenues for addressing hypoxia‐induced damage in aging tissues. Hypoxia preconditioning of stem cells has demonstrated potential in enhancing their regenerative capabilities, suggesting that modulating the hypoxic environment could improve outcomes in tissue repair and antiaging therapies.^306^, ^307^ Future research should explore the therapeutic potential of combining hypoxia‐modulating agents with regenerative approaches to enhance the efficacy of stem cell‐based treatments. Interdisciplinary collaboration, innovative clinical trial designs, and a commitment to ethical principles will be essential to advance the field and improve the health and well‐being of aging individuals. By unraveling the complex relationship between hypoxia and aging, we can pave the way for new therapeutic approaches that enhance healthy aging and reduce the burden of age‐related diseases. Continued research will be pivotal in unlocking the potential of hypoxia‐related interventions, ultimately improving the quality of life for aging populations worldwide.

## AUTHOR CONTRIBUTIONS

Ayesha Nisar and Sawar Khan performed the literature search, wrote the manuscript. Wen Li, Li Hu, Priyadarshani Nadeeshika Samarawickrama, Naheemat Modupeola Gold, Meiting Zi, Sardar Azhar Mehmood, Jiarong Miao, and Yonghan He revised the manuscript. Yonghan He conceived and supervised the review, wrote and revised the manuscript. All the authors have read and approved the final version of the manuscript.

## CONFLICT OF INTEREST STATEMENT

The authors declare no conflicts of interest.

## ETHICS STATEMENT

Not applicable.

## Data Availability

Not applicable.
